# Radiographic estimation of the growth rate of initially underdiagnosed ameloblastomas

**DOI:** 10.4317/medoral.23003

**Published:** 2019-06-25

**Authors:** Bruno ALA. Mariz, Bruno AB. Andrade, Michelle Agostini, Oslei-Paes de Almeida, Mário J. Romañach, Jacks Jorge Jr, Pablo A. Vargas, Marcio A. Lopes, Alan R. Santos-Silva, André-Caroli Rocha

**Affiliations:** 1 DDS, MSc. Oral Pathology, Department of Oral Diagnosis, Piracicaba Dental School, University of Campinas (UNICAMP), Piracicaba, Brazil; 2 DDS, MSc, PhD. Department of Oral Diagnosis and Pathology, Federal University of Rio de Janeiro (UFRJ), Rio de Janeiro, Brazil; 3 DDS, MSc, PhD. Oral Pathology, Department of Oral Diagnosis, Piracicaba Dental School, University of Campinas (UNICAMP), Piracicaba, Brazil; 4 DDS, MSc, PhD. Clinics Hospital of the Medical School, University of São Paulo (USP), São Paulo, Brazil

## Abstract

**Background:**

To evaluate the specific growth rate (SGR) of ameloblastoma.

**Material and Methods:**

Cases of ameloblastoma initially underdiagnosed (e.g. cases overlooked or diagnosed as reactive lesions) which had adequate radiographic documentation to evaluate their progression were retrospectively selected. Two panoramic radiographs were analyzed to determine the specific growth rate (SGR) of each tumor, defined as the logarithm of the ratio of final tumor area (when the diagnosis of ameloblastoma was made) to the initial tumor area (when the lesion was underdiagnosed), divided by the time interval between the radiographic images. The tumor area was measured using the software ImageJ.

**Results:**

Twelve patients with mandibular ameloblastomas were selected, including 5 males and 7 females, with a mean age of 24.9 years (range: 14-61 years). In four cases, the lesion was associated with the crown of an impacted third molar. In three cases, it was initially diagnosed as a periapical lesion. Three cases were extrafollicular and were not noticed in the initial radiographs. Two cases were initially diagnosed as ameloblastoma, but the surgery was delayed for personal reasons. The mean interval of time between the two radiographic images was 4.3 years (range: 0.4-9 years). Based on our analysis, ameloblastoma grows in average 40.4% per year (range: 14.9-88.7%).

**Conclusions:**

Ameloblastoma is a progressively growing tumor, but its growth rate seems to be smaller than initially reported in the literature. Better understanding the radiographic progression of ameloblastoma might improve its early diagnosis, management, and prognosis.

** Key words:**Ameloblastoma, odontogenic tumors, growth rate, panoramic radiograph.

## Introduction

Ameloblastoma is the most common odontogenic tumor, which is characterized by progressive growth, bone expansion and local recurrence (1-3). Ameloblastoma usually occurs as unilocular or multilocular radiolucencies in the posterior region of the mandible of adults, with no sex predilection and a wide age range (3,4).

Clinically, asymptomatic swelling in the involved area is the main complaint, but lesions may be occasionally painful (5,6). Besides, duration of symptoms until diagnosis varies considerably (3), and patients’ perception of symptoms might not represent the natural history of ameloblastoma (6).

Currently, there is limited data in the literature exploring the growth rate and time of progression of ameloblastoma (6-8). Therefore, we aimed to estimate the growth of ameloblastoma based on its early radiographic manifestations.

## Material and Methods

Cases of ameloblastoma were retrospectively selected from the files of Clinics Hospital of the Medical School of the University of São Paulo (USP) São Paulo, Brazil, Federal University of Rio de Janeiro (UFRJ) Rio de Janeiro-Brazil, and Piracicaba Dental School, University of Campinas (UNICAMP), São Paulo, Brazil in the period from 1999 to 2018.

-Inclusion criteria 

To evaluate ameloblastoma progression, cases with at least two panoramic images – one immediately prior to the diagnosis (final) and another panoramic radiograph where the lesion already could be seen, but it was initially underdiagnosed (initial). In the initial radiographs, the lesions were either overlooked, or diagnosed as periapical lesions, dentigerous cysts, or enlarged dental follicles.

-Exclusion criteria

Cases with incomplete clinical data, insufficient radiographic documentation or previous surgery were excluded from this study.

Specific radiographic growth rate assessment 

The tumor area in both radiographic images of each case included in the study was measured by software ImageJ (version 1.51j8, National Institutes of Health, USA) (9). For each case, the mesial-distal distance of a molar adjacent to the lesion was measured in both radiograph images using the “straight line” tool. This distance was used to calibrate the measurement tool. The “polygon selection” tool was then used to calculate the virtual area (initial and final) of the lesions. To determine the specific growth rate (SGR; growth % per year) of ameloblastoma, the formula described by Mehrara *et al.* (10) to quantify tumor response to antineoplastic treatment was adapted to our study. Hence, the logarithm of the ratio of final tumor area (A2) to the initial tumor area (A1) was divided by the time interval between the radiographic images (T2 - T1), as follows: SGR = ln(A2/A1)/T2 – T1.

Consequently, an estimated growth percentage per year was calculated for patients with ameloblastoma. This study was approved by the local Institutional Ethics Committee, under the protocol 03586418.4.0000.5418, following the Helsinki Declaration for studies involving human subjects.

## Results

Twelve cases fulfilled our inclusion criteria. There were 5 males and 7 females, with a mean age of 24.9 years (range: 14-61 years). All tumors occurred in the mandible, eleven of them in the posterior region, with a single case affecting the anterior region. The mean interval of time between the two radiographic images was 4.3 years (range: 0.42-9 years). Based on our analysis, mean SGR was 40.4% per year (range: 14.9-88.7%), as showed in  Table 1. In four cases (5, 6, 8, and 11), the ameloblastoma was associated with the crown of an unerupted third molar, and in three cases (2, 9, and 12), the tumor was misdiagnosed at first as a periapical lesion. Three cases (1, 3, and 4) were extrafollicular, and were not noticed in the initial radiographs. Cases 7 and 10 were initially diagnosed as ameloblastoma, but the surgery was delayed for personal reasons. Eleven cases were microscopically diagnosed as multicystic ameloblastomas, and one case (case 9) as unicystic ameloblastoma.

**Table 1:**
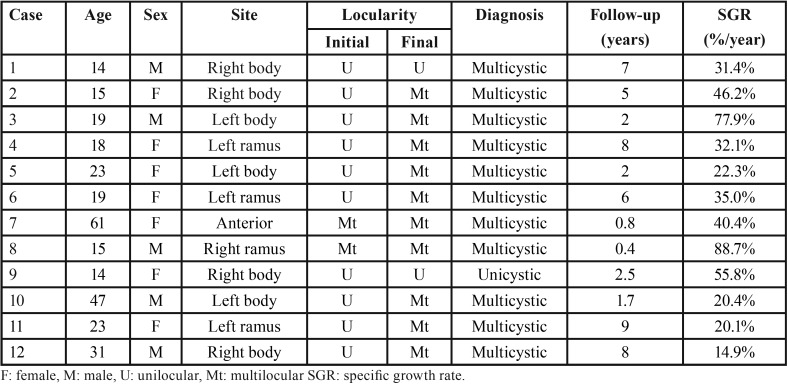
Clinical features and specific growth rate (SGR) of 12 cases of ameloblastoma.

**Figure 1:**
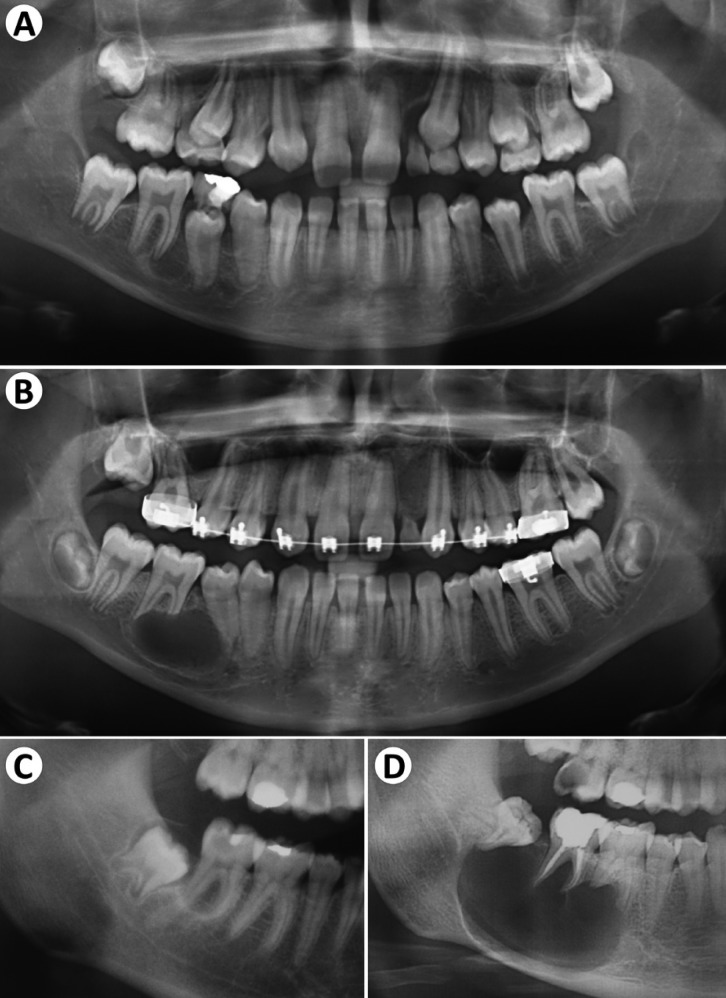
Progression of Ameloblastoma seen in cases 9 (A-B) and 2 (C-D). (A) a radiolucent image is seen associated with the mesial root of the right inferior first molar. (B) After 2.5 years of orthodontic treatment, the lesion increased in size, causing root displacement and resorption. (C) A small radiolucent image is seen associated with the right inferior second molar. (D) After 5 years, the tooth was endodontically treated, but the lesion significantly progressed, distally displacing the third molar.

**Figure 2:**
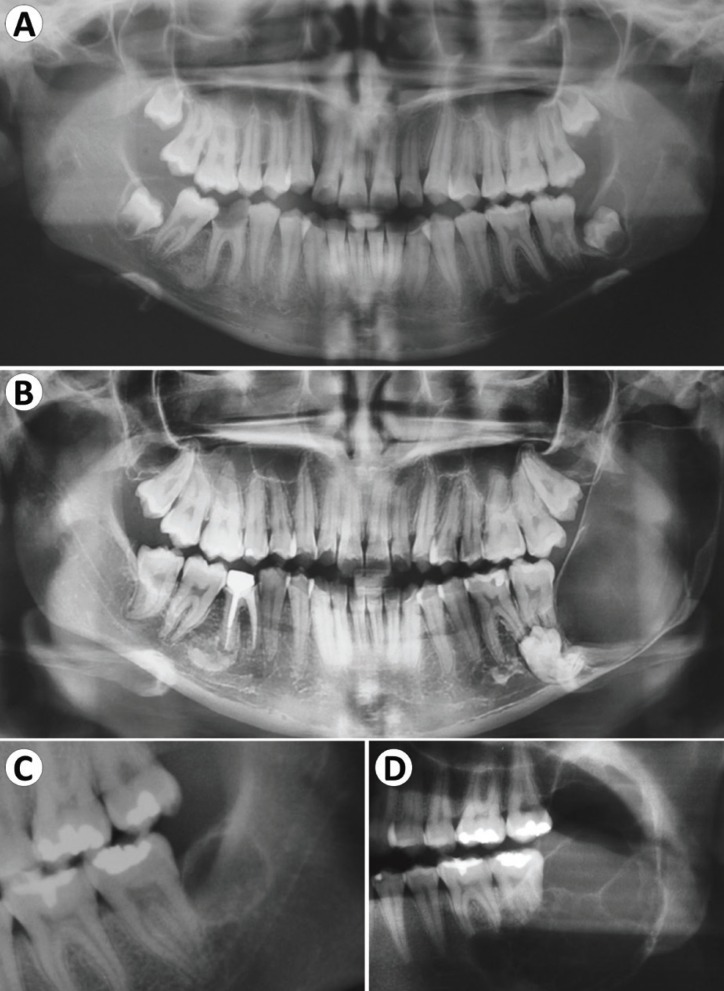
Progression of Ameloblastoma seen in cases 6 (A-B) and 4 (C-D). (A) Small radiolucent images are seen around the inferior third molars. (B) Note the normal eruption of the right inferior third molar, while a radiolucent image occupies the left ramus after 6 years. (C) A small round radiolucent image is seen distally to the left inferior second molar. (D) After 8 years, the lesion increased significantly, occupying part of ramus and body of the mandible.

Representative images of four of the cases are presented below (Figs. 1,2). In case 9, a 14-year-old male presented a well-defined radiolucency causing root resorption of the right lower first molar noticed during evaluation of a panoramic radiograph acquired during orthodontic treatment. The panoramic radiograph acquired 2.5 years earlier showed a smaller radiolucency between the right lower second premolar and first molar (Fig. 1A-B). In case 2, a 15-year-old female presented a small well-defined radiolucency associated with the right lower second molar. After 5 years, the tooth had been endodontically treated, but the lesion significantly progressed, displacing distally the third molar and inferiorly the mandibular canal (Fig. 1C-D).

In case 6, a 19-year-old female was diagnosed with an expansile ameloblastoma in the left ramus of the mandible causing displacement of the third molar and the mandibular canal. The patient had another panoramic radiograph taken 6 years prior to the diagnosis, where small radiolucencies were observed around the crown of both lower third molars, compatible with enlarged dental follicles/dentigerous cysts. Evaluating the current radiograph, the right third molar erupted normally, while the tumor developed in the opposite site (Fig. 2A-B). In case 4, an 18-year-old female presented a small, round and unilocular radiolucency located distally to the left inferior second molar, replacing the third molar, which is absent. After 8 years, the lesion increased significantly, assumed a multilocular aspect, expanding the bone cortices of the posterior mandible and causing root resorption of the second molar (Fig. 2C,D).

## Discussion

Ameloblastoma is a progressively growing benign odontogenic tumor characterized by its aggressive behavior and local recurrence (1). Nearly 90% of ameloblastomas present mutations in genes related to the MAPK pathway (11), specifically the BRAF V600E, which has diagnostic and prognostic implications (12,13).

However, few studies have quantified ameloblastoma growth rate (6-8). Odukoya & Effiom (7) analyzed one hundred cases of ameloblastoma and correlated the estimated volume of the tumor with the duration of symptoms, estimating a growth rate of 0.81cm3/month (9.72 cm3/year) for conventional ameloblastoma, and of 0.17cm3/month (2.04 cm3/year) for unicystic ameloblastoma. Similarly, Effiom & Odukoya (8) evaluated the largest diameter of ameloblastomas and compared it with the duration of the tumor (in months). Thereby, the authors estimated that conventional ameloblastomas grew an average of 0.7 cm/month. Despite these studies had added valuable information to better understand the clinical progression of ameloblastoma, they used the duration of symptoms reported by patients during consultation to calculate the tumors’ growth rate, which may not represent an accurate measure of time.

Interestingly, Chae *et al.* (6) attempted to establish the SGR of ameloblastoma in a systematic review with meta-analysis, estimating a mean SGR of 87.8% per year. The authors searched the literature for case reports or case series of ameloblastoma where all three dimensions and the duration of symptoms of the tumor were reported. Sixteen studies were selected, including 14 reports of single cases and 2 case series (14-25). However, these studies do not have a previous documentation of any case. Once more, the time period used in all analyses is based on patients’ perception of symptoms, which may not be completely exact. Furthermore, since the authors did not have an initial measure to calculate the SGR of the tumors, they simply divided the final measure by 1, which could further distort this analysis.

In addition, Pereira-Castro-Lopes *et al.* (26) reported a case of a 20-year-old male presenting a significant ameloblastoma lesion occupying the right body and ramus of the mandible, causing tooth resorption. The patient had another radiograph taken 4 years before for orthodontic planning purposes. A circumscribed unilocular image could already be observed in association with an unerupted third molar. Applying the same formula used in our study, an SGR of 29.5%/year was calculated, which is in accordance with our results.

We found a smaller SGR (40.4%/year) compared to the study of Chae *et al.* (6) (87.8%/year), which might be explained by a delayed perception of symptoms by patients, since the lesion could be present long before the first symptom, thereby inflating the SGR calculated. The highest SGR values (88.7% and 77.9% growth per year) in our series occurred in young males (15 and 19-year-old, respectively) with small intervals of time between radiographs (0.42 and 2 years, respectively), which could indicate that ameloblastoma may exhibit an accelerated growth in determined periods of time.

Remarkably, three of our cases were initially diagnosed as periapical lesions, reinforcing that careful evaluation of clinical, radiographic and microscopic features is imperative to diagnose ameloblastoma, avoiding unnecessary endodontic treatment, therefore delaying proper management (27). Furthermore, another two cases were initially considered as dentigerous cysts or enlarged dental follicles. Hence, it seems reasonable to maintain close follow-up for lesions that might rise suspicion of ameloblastoma, and a biopsy must be performed to stablish a definite diagnosis.

We acknowledge the fact that panoramic radiographs depict a bidimensional representation of the real tumor, and these images are subjected to distortion, which may not reflect the real size of a lesion (28). Moreover, computed tomography (CT) and magnetic resonance imaging (MRI) analyses would not only contribute to evaluate ameloblastoma progression, but also aid in distinguishing ameloblastomas from other cyst like lesions (29,30). However, considering the lack of solid evidence in the literature, we believe that the analysis performed, despite simple, might improve the current understanding about ameloblastoma natural progression when not treated.

In summary, we estimate that ameloblastoma has a mean growth rate of 40.4% per year, which represents a considerably smaller percentage than previous reported in the literature. Better understanding the radiographic progression of ameloblastoma might improve its early diagnosis, management, and prognosis.
